# The Increased FCRL mRNA Expression in Patients with Graves’ Disease Is Associated with Hyperthyroidism (But Not with Positive Thyroid Antibodies)

**DOI:** 10.3390/jcm13175289

**Published:** 2024-09-06

**Authors:** Katarzyna Wojciechowska-Durczynska, Jan Stepniak, Andrzej Lewinski, Malgorzata Karbownik-Lewinska

**Affiliations:** 1 Department of Endocrinology and Metabolic Diseases, Medical University of Lodz, 93-338 Lodz, Poland; katarzyna.wojciechowska-durczynska@umed.lodz.pl (K.W.-D.); jan.stepniak@umed.lodz.pl (J.S.); 2 Department of Endocrinology and Metabolic Diseases, Polish Mother’s Memorial Hospital-Research Institute, 93-338 Lodz, Poland; andrzej.lewinski@umed.lodz.pl

**Keywords:** FCRL, Graves’ disease, Graves’ orbitopathy, hyperthyroidism, autoimmunity

## Abstract

**Background:** Fc receptor-like (FCRL) genes play a role in the immune system by encoding proteins that function as receptors on the surface of immune cells. The clinical significance of FCRL gene expression in Graves’ Disease (GD) and Graves’ Orbitopathy (GO) remains unclear. We evaluated the expression of FCRL 2, 3, 4 mRNA in patients with GD and GO and its role in the development and activity of these diseases. **Methods:** Peripheral blood samples from patients with GD (*n* = 24) or GO (*n* = 49) hospitalized in the Department of Endocrinology and Metabolic Diseases, Medical University of Lodz, were collected. Expressions of FCRL2, FCRL3 and FCRL4 were measured by real-time PCR. **Results**: FCRL3 expression was higher in patients with GD compared to GO (1.375 vs. 0.673, *p* = 0.004) and, specifically, active GO (1.375 vs. 0.639, *p* = 0.005). Regarding FCRL4, mRNA expression was higher in GD compared to Control (3.078 vs. 0.916, *p* = 0.003), GO (3.078 vs. 1.178, *p* < 0.001), active GO (3.078 vs. 1.186, *p* = 0.002) and inactive GO (3.078 vs. 1.171, *p* = 0.008). In turn, FCRL4 mRNA expression was higher in patients with hyperthyroidism (subclinical + overt) than in euthyroid patients (2.509 vs. 0.995, *p* = 0.001 when the whole group of individuals was considered; 2.509 vs. 1.073, *p* = 0.004 when GO + GD was considered). **Conclusions:** The increased FCRL mRNA expression in patients with GD is associated with hyperthyroidism (but not with positive TSHRAbs), and our study is the first one to confirm this relationship.

## 1. Introduction

Graves’ Disease (GD) is an autoimmune disease caused by the presence of antibodies against thyroid-stimulating hormone receptors (TSHRAbs), usually characterized by hyperthyroidism. Graves’ Orbitopathy (GO) is the most common extra-thyroidal manifestation of GD, resulting from inflammation and proliferation of connective and adipose tissues in the orbit, stimulated by TSHRAbs and cytokines released by lymphocytes [1].

Fc receptor is a molecule expressed on the surface of immune cells. Its name is derived from a part of immunoglobulin known as the Fc (fragment crystallizable) region. Fc receptors recognize the Fc region of several immunoglobulin (Ig) classes, namely IgG, IgE, IgM, and IgA [2].

Fc receptor-like (FCRL) genes are a family of genes encoding molecules that function as receptors on the surface of immune cells, especially B-cells [3,4]. FCRL genes are located on chromosome 1, in close proximity to the Fc receptor gene. In humans, this gene family encodes six transmembrane receptors, i.e., FCRL1–6, and two intracellular proteins, i.e., FCRLA and FCRLB [4,5,6].

The biological role of FRCLs is not completely understood, but these molecules share many similar features with the classic Fc receptors [5,7]. FCRLs are known to interact with antibodies and other molecules involved in the immune response [6].

FCRL1-5 molecules are variably expressed by B lineage cells according to their stages of maturation and tissue localization. FCRL2 is identified mainly in tonsillar memory B-cells, whereas FCRL3 is found on splenic B-cells, NK-cells and regulatory T-cells. In turn, FCRL4 is present on memory B-cells in mucosa associated lymphoid tissues. Moreover, FCRL2 is present on peripheral blood memory B-cells in contrast to FCRL3 and FCRL4, the latter being rarely found in blood from healthy individuals [5,6].

**Figure 1:**
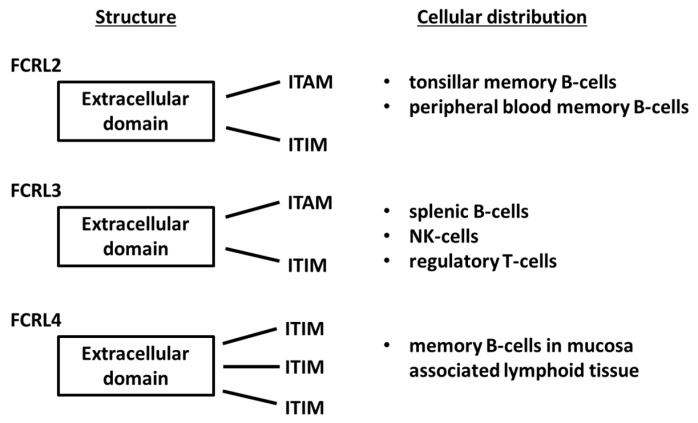
Structure and cellular distribution of FCRL isoforms, prepared on the basis of data found in the literature [5,6,9]. ITAM—immunoreceptor tyrosine-based activation motif; ITIM—immunoreceptor tyrosine-based inhibitory motif.

FCRL proteins as the transmembrane receptors have extracellular domain and intracellular domain which contains immunoreceptor tyrosine-based activation motifs (ITAMs) and/or immunoreceptor tyrosine-based inhibitory motifs (ITIMs). Both activating and inhibitory proteins—by crosstalk with B-cell receptors (BCRs)—can transmit signals inside B-cells. In that way, FCRL proteins can affect B-cell function [8]. The intracellular domain of FCRL4 contains three ITIMs, which theoretically suggests only the inhibitory potential, whereas FCRL2,3 contain both ITAM and ITIM, which suggests the potential for activating and inhibitory functions [6]. However, this dual potential may also concern FCRL4, because its ITIM possesses a switching ability, i.e., it possesses an immunoreceptor tyrosine-based switch motif (ITSM) [9]. Moreover, FCRL4 ITIM could form a canonical ITAM in cooperation with neighboring ITIMs [6]. FCRL structures and cellular distributions are illustrated in Figure 1.

It has been shown in the earlier study that the pathological activation of some FCRL molecule expression leads to the down regulation of BCR-mediated signaling, incomplete anergy and deletion in autoreactive B-cells and, finally, the breakdown of B-cell tolerance [4,10]. Therefore, FCRL molecules were considered to play a role in the pathogenesis of autoimmune diseases. FCRL3 has been confirmed to inhibit BCR signaling when it is overexpressed [10]. Similarly, a negative effect of FCRL2 and FCRL4 overexpression on BCR signaling was observed [6].

In the earlier study, overexpression of FCRL2 and FCRL4 was observed in patients with GD but without an association with thyroid peroxidase antibodies (TPOAbs) or thyroglobulin antibodies (TgAbs) [11]. The increased expression of the FCRL3 mRNA level, as a result of single nucleotide polymorphism (SNP), was also confirmed in GD [12]. Nonetheless, the published evidence on clinical significance of FCRL expression in GD remains unclear, and, in regard to GO, it has never been examined, and our study does constitute a step to broaden this field.

The aim of the present study was to evaluate the expression of FCRL 2, 3, 4 mRNA in patients with GD and GO and its role in the development and activity of these diseases. Also, a possible relationship between FCRL mRNA expression and routinely evaluated laboratory parameters, which may be affected by GD and GO, was examined.

## 2. Materials and Methods

The Ethical Committee of the Polish Mother’s Memorial Hospital—Research Institute, Lodz, Poland, approved all the procedures applied in the present study, and fully informed written consent was obtained from all patients (No. 40/2019; 19 March 2019).

Eighty-nine (89) individuals (75 females, 84%) aged 18–80 years who were patients of the Department of Endocrinology and Metabolic Diseases, Medical University of Lodz, Poland, were enrolled to the study in the years 2019–2022. Approximately half of patients with a diagnosis of GO and a few patients with a diagnosis of GD were recruited from our previous study [13].

The patients were divided into the following three main groups: Controls—sixteen (16) out of 89 subjects (16/89; 18%) who were healthy individuals and were confirmed to be individuals without a history of GD, GO or any other autoimmune disease; Patients with GD—twenty four (24) out of 89 subjects (24/89; 27%) with a positive test for a TSH receptor autoantibody (TSHRAb) and laboratory features of thyrotoxicosis; Patients with GO—forty nine (49) out of 89 subjects (49/89; 55%) in whom diagnosis of GO was based on standard criteria, including positive tests for TSHRAbs currently or in medical history and positive ophthalmic assessment. The third group of 49 patients was further divided into the two following groups: thirty-seven (37) out of 89 subjects (37/89; 42%) diagnosed with active GO and twelve (12) out of 89 subjects (12/89; 13%) with inactive GO. GO was classified as clinically active or inactive, as recommended by the European Group on Graves’ Orbitopathy (EUGOGO) [14]. All patients with active GO were qualified for further tests before administration of intravenous steroids. Therefore, none of the patients (either with GD or with GO) did obtain glucocorticoids (for any reasons). Most patients with GD were newly diagnosed with hyperthyroidism and, therefore, had not received any pharmacological treatment. Only a few GD patients had received short-term treatment with antithyroid medications, but they were still hyperthyroid. Regarding GO, most patients were receiving antithyroid drugs, with a few receiving Levothyroxine, and they were either euthyroid patients or had subclinical hyperthyroidism (slightly decreased TSH with FT3 and FT4 in reference ranges) when they were enrolled in the study.

The exclusion criteria constituted malignant diseases and severe acute/chronic diseases other than thyroid diseases. Body mass and body height were measured to calculate the BMI. Blood samples were collected after an overnight fast at 7 a.m., and all laboratory parameters were measured either in the whole blood or blood serum for diagnostic purposes. Additionally, EDTA blood samples (1 mL) were collected and stored at −80 °C until being used in an appropriate assay to measure the FCRL mRNA expression levels.

**Figure 2:**
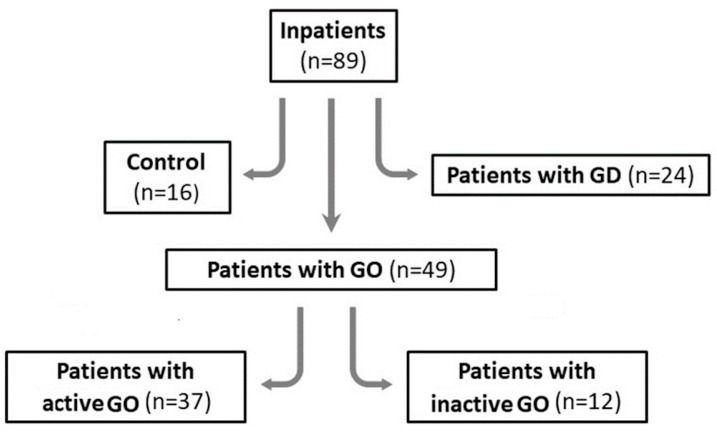
Study design diagram. GD, Graves’ Disease; GO, Graves’ Orbitopathy.

Study design diagram is presented in Figure 2.

### 2.1. Parameters Measured in Blood Serum

The immunochemiluminescent method (Cobas e-601; Roche Diagnostics, Mannheim, Germany) was used to measure the concentrations of hormones (thyroid-stimulating hormone, TSH; free triiodothyronine, FT3; free thyroxine, FT4), thyroid antibodies (thyroid peroxidase antibody, TPOAb; thyroglobulin antibody, TgAb; TSH receptor antibody, TSHRAb) and vitamin D in blood serum. The standard methods (Vitros 4600/Vitros 5.1; Johnson & Johnson, Beerse, Belgium) were used to measure other laboratory parameters (total cholesterol, TChol; HDL cholesterol, HDLC; LDL cholesterol, LDLC; triglycerides, TGs; glucose; aspartate aminotransferase, ASPAT; alanine aminotransferase, ALAT; C-reactive protein, CRP) in blood serum. A Sysmex XN-2000 Hematology System was used to measure the complete blood count, i.e., red blood cells, RBC; hemoglobin, Hgb; white blood cells, WBC; hematocrit, HCT; platelets; neutrophils; lymphocytes; eosinophils; basophils and monocytes. Anthropometric measurements included body height and body mass, which were used to calculate the body mass index (BMI).

### 2.2. Assay to Measure FCRLs mRNA Expression

Blood samples from patients without thyroid autoimmunity served as a control for a real-time PCR experiment (calibrator). The total RNA from the blood was extracted according to a modified Chomczynski and Sacchi’s method. The purity of the total RNA was assessed by NanoDrop^®^ ND-100 spectrophotometr (Nanodrop Tech, Wilmington, DE, USA). The total RNA was used in the first strand cDNA synthesis with TaqMan^®^ Reverse Transcription Reagents (Applied Biosystem, Branchburg, NJ, USA) according to manufacturers’ instruction. Real-time PCR was performed on the ABI PRISM^®^ 7500 Sequence Detection System (Applied Biosystem, Foster City, CA, USA) by using a TaqMan^®^ Universal PCR Master Mix (Applied Biosystem) and TaqMan^®^ Gene Expression Assays probe and primer mix (Applied Biosystem) according to the manufacturer’s specification. The Assays Identification numbers were FCRL2: Hs00937765_m1, FCRL3: Hs00364720_m1 and FCRL4: Hs00972783_m1. The thermal cycler conditions were as follows: hold for 10 min at 95 °C, followed by two-step PCR for 50 cycles of 95 °C for 15 s followed by 60 °C for 1 min. Amplification reactions, in triplicate for each sample, were performed, and the results were normalized to the GAPDH gene expression level. An analysis of relative gene expression data was performed using the 2^−ΔΔCT^ method on an ABI PRISM^®^ 7500 Sequence Detection System Software v2.0.6 (Applied Biosystems, Waltham, MA, USA). The calibrator was prepared as a cDNA mix from all cDNA samples. The fold change in studied gene expression, normalized to endogenous control, was calculated using RQ = 2^−ΔΔCT^. Results are presented in Figures as FCRL RQ.

### 2.3. Statistical Analyses

A Student’s unpaired *t*-test or the Mann–Whitney U test were used to compare mean or median values, respectively, of different clinical/laboratory parameters between independent groups. The results are presented as means ± SEM or median with 25th and 75th percentiles, respectively. Univariate regression analyses were performed to determine which linear (continuous) variables might have been associated with considered dichotomized variables (patients with hyperthyroidism vs. euthyroid patients; patients with active vs. inactive GO). Statistical analyses were performed using SigmaPlot version 11.0 (RRID: SCR_003210) (Systat Software, Inc., San Jose, CA, USA). Statistical significance has been determined at the level of *p* < 0.05.

## 3. Results

Linear clinical/laboratory parameters in control individuals (*n* = 16) were compared to those obtained in patients with Graves’ Disease (GD; *n* = 24), in patients with Graves’ Orbitopathy (GO; *n* = 49), as well as specifically in patients with active Graves’ Orbitopathy (active GO; *n* = 37) and with inactive Graves’ Orbitopathy (inactive GO; *n* = 12) (Table 1).

Patients with GO and active GO were, unfortunately, older than healthy individuals; however, no differences were recorded between the examined groups. Regarding body mass and BMI, individual examined groups did not differ statistically compared to healthy individuals; however, patients with GD have a lower body mass and BMI comparing to patients with GO (*p* = 0.050; *p* = 0.035, respectively), patients with inactive GO (*p* = 0.004; *p* = 0.003, respectively) and patients with active GO (*p* = 0.025; *p* = 0.021, respectively).

As expected, the TSH concentration was lower and FT3 and FT4 concentrations were higher in patients with GD compared to control individuals. Also, the TSH was lower in patients with GD vs. patients with inactive GO (*p* = 0.043), and FT3 and FT4 were higher in patients with GD vs. patients with GO (*p* < 0.001, *p* < 0.001, respectively), patients with active GO (*p* < 0.001, *p* < 0.001, respectively) and patients with inactive GO (*p* = 0.006, *p* = 0.013, respectively).

Concentrations of thyroid antibodies were generally higher in examined groups than in Controls, and the precise results are as follows. TPOAbs were significantly higher in examined groups, i.e., patients with GD, patients with GO, patients with active GO and patients with inactive GO, compared to Control individuals (Table 1). At the same time, TPOAb concentrations did not differ significantly between individual examined groups.

Regarding TgAb concentrations, although mean values were much higher in examined groups than in control individuals, these differences did not reach statistical significance. At the same time TgAb concentrations did not differ significantly between individual examined groups.

In turn, TSHRAbs were significantly higher in examined groups, i.e., patients with GD, patients with GO, patients with active GO and patients with inactive GO, compared to control individuals (Table 1). At the same time, TSHRAb concentrations did not differ significantly between individual examined groups.

Slightly lower (although statistically significant) hemoglobin (Hgb) concentrations in patients with GO, patients with active GO and in patients with inactive GO vs. healthy individuals (Table 1) is not of scientific significance (it can result from a bias); thus, it can be neglected and will not be discussed in the present work.

Because the main parameter measured in the current study, i.e., FCRL molecule, is localized on the surface of circulating lymphocytes in peripheral blood, potential differences between individual groups regarding white blood cell (WBC) and lymphocyte concentrations should be considered. However, no statically significant differences were found in either WBC concentrations or lymphocyte concentrations between any two groups.

As hyperthyroidism can decrease neutrophil concentrations, it should be mentioned that neutrophil level in patients with GD (all of them were hyperthyroid) was not statistically lower compared to Control individuals; the same relates to patients with GO, patients with active GO and patients with inactive GO vs. control individuals (Table 1).

Regarding lipid profile, lipid fractions, such as TChol, HDLC, LDLC and TGs, did not differ significantly between examined groups, i.e., patients with GD or patients with GO or patients with active GO or inactive GO, vs. Control individuals (Table 1). Regarding C-reactive protein (CRP), it did not differ statistically between any two examined groups.

**Figure 3:**
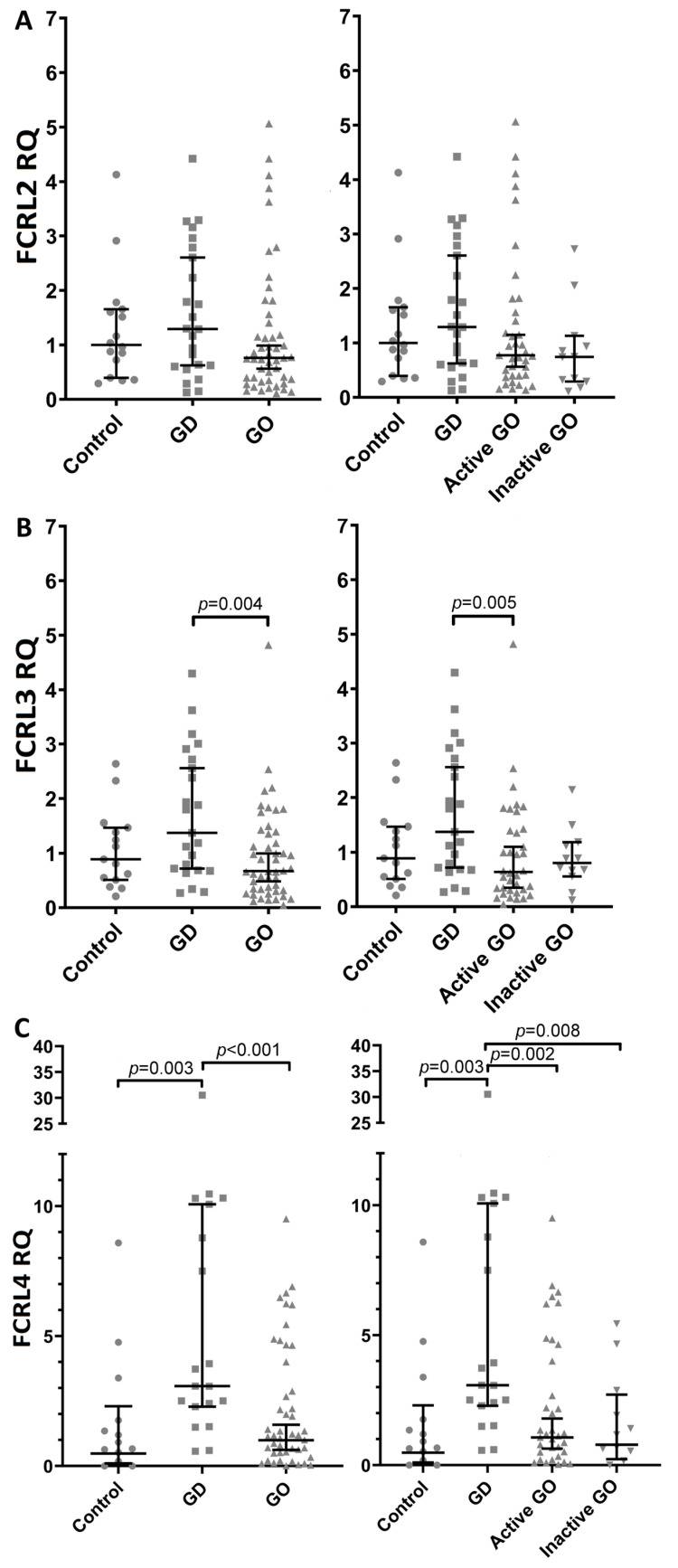
Median of relative mRNA expression (RQ) of particular FCRL subtypes ((**A**) FCRL2, (**B**) FCRL3, (**C**) FCRL4) in Control individuals (*n* = 16), in patients with GD (*n* = 24), in all patients with GO (*n* = 49) (left graphs) and also, separately, in patients with active GO (*n* = 37) and in patients with inactive GO (*n* = 12) (right graphs). Statistical evaluation was performed by Mann–Whitney test. Black horizontal lines represent median values (longer lines) and the 25th and 75th percentiles (shorter lines) The statistical differences are marked in the graphics. Statistical significance was determined at the level of *p* < 0.05. Explanation of symbols in Figure: dot—Control, square—GO, triangle—GO or active GO, inverted triangle—inactive GO.

Relative mRNA expression levels of particular FCRL subtypes (FCRL2, FCRL3, FCRL4) in patients with GD, patients with GO and patients with active GO and inactive GO are presented in Figure 3. Regarding FCRL2 mRNA expression, although its median value was slightly higher in patients with GD vs. control individuals (1.293 vs. 1.001 *p* = 0.492) (Figure 3A left and right) vs. patients with GO (1.293 vs. 0.761 *p* = 0.101) (Figure 3A left), vs. patients with active GO (1.293 vs. 0.772 *p* = 0.222) (Figure 3A right) and vs. patients with inactive GO (1.293 vs. 0.743 *p* = 0.062) (Figure 3A right), no statistically significant differences were found with borderline significance between the GD group and the inactive GO group (*p* = 0.062).

FCRL3 mRNA expression was higher in peripheral blood from patients with GD compared to GO (1.375 vs. 0.673, *p* = 0.004) (Figure 3B left) as well as compared specifically to active GO (1.375 vs. 0.639, *p* = 0.005) (Figure 3B right). The difference between patients with GD and patients with inactive GO was of borderline significance (*p* = 0.062).

Regarding FCRL4 mRNA expression, an even more pronounced increase (comparing to FCRL3 mRNA expression) was found in GD patients. Namely, FCRL4 mRNA expression was higher in individuals with GD compared to Control (3.078 vs. 0.916, *p* = 0.003) (Figure 3C left and right), GO patients (3.078 vs. 1.178, *p* < 0.001) (Figure 3C left), active GO patients (3.078 vs. 1.186, *p* = 0.002) (Figure 3C right) and inactive GO patients (3.078 vs. 1.171, *p* = 0.008) (Figure 3C right). After one patient with GD was removed from statistical analysis due to very high FCRL4 RQ (30.531), statistical significance was still maintained and the results were as follows: FCRL4 mRNA expression was higher in GD patients compared to Control (3.073 vs. 0.916, *p* = 0.007), GO patients (3.073 vs. 1.178, *p* = 0.002), active GO patients (3.073 vs. 1.186, *p* = 0.005) and inactive GO patients (3.073 vs. 1.171, *p* = 0.014).

Of significance was to consider if the increased expression of FCRL mRNA is associated with active/inactive GO. Therefore, we have performed the following statistical analyses.

Regarding FCRL3 mRNA expression, no statistically significant differences were found between active GO and inactive GO (0.639 vs. 0.803, *p* = 0.362) as well as between Controls and active GO (0.889 vs. 0.639, *p* = 0.968) or inactive GO patients (0.889 vs. 0.803, *p* = 0.456).

When FCRL4 mRNA expression was analyzed, similarly, no statistically significant differences were found between active GO and inactive GO patients (1.186 vs. 1.171, *p* = 0.410) as well as between Controls and active GO (0.916 vs. 1.186, *p* = 0.729) or inactive GO (0.916 vs. 1.171, *p* = 0.232).

Next, the univariate logistic regression analysis was performed to check if any of the FCRL mRNA expressions, as linear variables, are associated with active GO or inactive GO (Table 2). No statistically significant association was found with active/inactive GO for any considered linear parameters (FCRL2, FCRL3, FCRL4) in univariate regression analysis.

**Figure 4:**
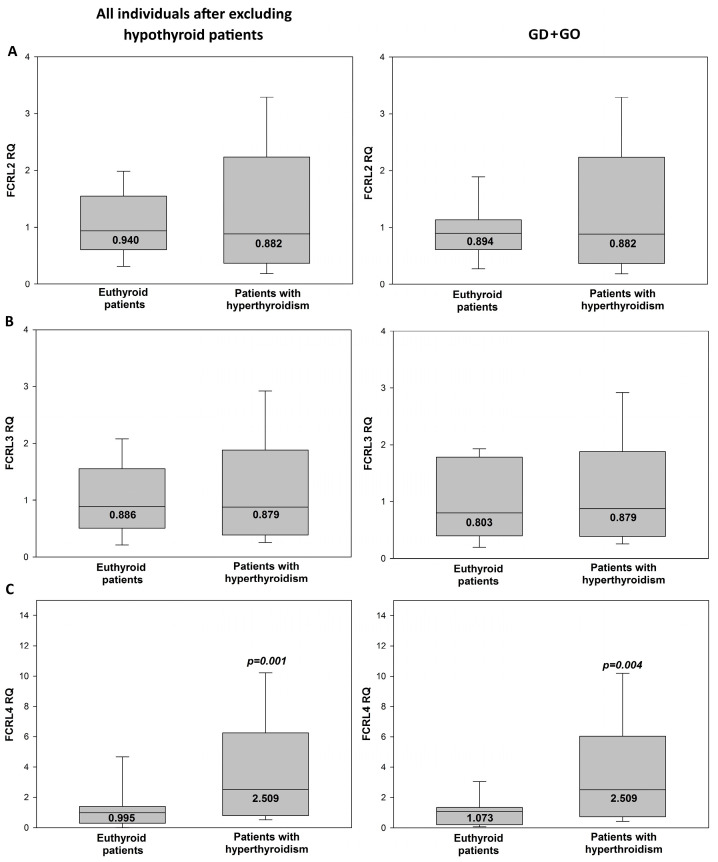
Median of relative mRNA expression of particular FCRL subtypes ((**A**) FCRL2, (**B**) FCRL3, (**C**) FCRL4) in the whole group of individuals (after excluding hypothyroid patients; *n* = 82) divided into euthyroid (*n* = 32) and hyperthyroid (*n* = 50) patients (left graphs), and in the group of patients with GD + GO (*n* = 66) divided into euthyroid (*n* = 16) and hyperthyroid (*n* = 50) patients (right graphs). Statistical evaluation was performed by a Mann–Whitney test. Black horizontal lines within the box represent median values, the upper and lower boundaries of the box mark the 25th and 75th percentiles and the whiskers mark the minimum and maximum values. The statistical differences are marked in the graphics. Statistical significance was determined at the level of *p* < 0.05.

In turn, mRNA expression levels of particular subtypes of FCRLs were compared between patients with hyperthyroidism (subclinical + overt) and euthyroid patients, when the whole group of individuals were considered (however, after excluding hypothyroid patients; *n* = 89 − 7 = 82) (Figure 4 left). The same was analyzed in the group of GD + GO patients (*n* = 24 + 42 = 66). Regarding FCRL2 or FCRL3 mRNA expression levels, they did not differ between hyperthyroid and euthyroid patients (Figure 4A,B, left and right). Instead, FCRL4 mRNA expression was higher in patients with hyperthyroidism (subclinical + overt) than in euthyroid patients (2.509 vs. 0.995, *p* = 0.001) when the whole group of individuals was considered (Figure 4C, left) as well as when only GO + GD was considered (2.509 vs. 1.073, *p* = 0.004) (Figure 4C, right).

To determine which linear variables are associated with hyperthyroidism, a univariate logistic regression analysis was performed (Table 3). The regression analysis was performed in the following groups: the whole group of individuals (*n* = 89) (Table 3, above) and the whole group of individuals after excluding hypothyroid patients (*n* = 89 − 7 = 82) (Table 3, below). Among the three subtypes of FCRLs, the only variable associated with hyperthyroidism was FCRL4 mRNA expression (Table 3, above and below). Thus, the positive association of FCRL4 mRNA expression with hyperthyroidism described above (Figure 3) was confirmed in the univariate regression analysis. No statistically significant association was found for any other considered linear parameter in the univariate regression analysis. Such variables as thyroid tests (TSH, FT3 and FT4) and thyroid antibodies (TSHRAbs, TPOAbs, TgAbs) were not put into this statistical analysis, as they are directly associated with a dichotomized variable, i.e., hyperthyroidism.

To evaluate possible associations of FCRL expression with thyroid antibodies, we have checked if medians of relative mRNA expression of particular FCRL subtypes (FCRL2, FCRL3, FCRL4) differ between patients with positive vs. negative thyroid antibodies (TPOAb (+) vs. TPOAb (−); TgAb (+) vs. TgAb (−); TSHRAb (+) vs. TSHRAb (−)). Such comparisons were conducted in the group of patients with GD, in the group of patients with GO and in the group comprising patients with either GD or GO (GD + GO) (Table 4). No statistically significant differences in FCRLs mRNA expression levels were found between patients with positive vs. negative thyroid antibodies.

The lack of above association between FCRLs expression and thyroid antibodies was confirmed by univariate regression analysis (Table 5). Namely, expression of none of the FCRL subtypes was significantly associated with positive TPOAbs, positive TgAbs or with positive TSHRAbs (Table 5).

## 4. Discussion

Pathogenesis of GD is a matter of multiple susceptibility genes, and the occurrence may be predisposed by smoking, infections and stress. The environmental and internal factors can together activate T-cells and B-cells to respond to thyroid autoantigen, leading to development of GD and GO [15,16,17].

FCRL expression patterns had been studied in autoimmune thyroid diseases and provide some controversial and scanty results. Rostamzadeh et al. (2015) observed up-regulation of FCRL2 mRNA expression not only in GD but also in Hashimoto thyroiditis (HT) patients compared to healthy individuals, as well as the increased FCRL4 mRNA expression in GD patients (similarly to our results) but not in HT patients [11].

In turn, elevated expression of FCRL3 was proved in long-standing and/or more aggressive forms of HT [18]. Furthermore, it has been identified in our previous study that FCRL3 is overexpressed in patients with HT, that FCRL3 expression can be associated with patients’ age and that it is significantly higher in children compared to adults with HT [19].

Regarding FCRL3 polymorphism, it has been revealed that it is related to GD with regional and ethnic variability; however, no significant differences in age of onset, gender or severity of goiter were found between the studied genotypes [15]. In agreement with our results, it was demonstrated that certain SNPs of FCRL3 lead to increased expression of FCRL3 mRNA levels in GD [12]. It has also been presented that the FCRL3 polymorphisms are related to the high risk of GO in Asian populations (Chinese and Japanese), while contradictory results remained in Caucasian populations [20].

It should be emphasized that, until now, no studies on FCRL mRNA expression and its potential role in GO have been conducted. Because GO is one of the most common complications of GD, we assumed that the FCRL expression in GD and in GO would be similar and that any changes in these expressions would be analogous. However, our results confirmed the increased FCRL3 and FCRL4 mRNA expression only in patients with GD but not in patients with GO, and our findings did not show any association between FCRL2, FCRL3 as well as FCRL4 mRNA expression levels and the presence of active or inactive GO. However, keeping in mind that there are also multiple FCRL protein isoforms resulting from alternative splicing of each individual FCRL mRNA, and that these structural differences in FCRL protein could influence FCRL ligand binding and signaling [21], it is probable that there could be differences in expression at the level of FCRLs protein isoforms in patients with active and inactive GO, and this requires special attention in the context of further studies.

Actually, the occurrence of GO has been demonstrated as a consequence of cumulative effects of both genetic and environmental factors, with the precise mechanism still not being known [16,17]. On the basis of our results, we can only presume that FCRLs do not contribute substantially to the activity of the process of GO. Our previous study showed an association between reduced numbers of dendritic cells (DCs) in peripheral blood in patients with GO; therefore, it is more likely that DCs, instead of FCRL positive B-cells, contribute to the pathogenesis of GO [13].

The most important observation of the present study is that FCRL3 and FCRL4 expression levels were increased in GD patients compared to patients with GO and compared to healthy individuals. Therefore, we have focused our considerations on potential mechanisms of the above findings. Two main potential mechanisms which we took into account were autoimmune process and thyroid dysfunction.

Regarding the first mechanism, i.e., the possible autoimmune disturbances, there are some discrepancies regarding the relationship between antithyroid antibodies and the FCRL genes. In an earlier study, FCRL2 and FCRL4 gene expression levels showed no correlation with the levels of TPOAbs and TgAbs from GD and HT patients [11]; however—in another study—FCRL3 expression positively correlated with TPOAb levels in HT [18]. While FCRL3 polymorphism was associated with positive TSHRAbs in patients with GD, no relationship was found with TPOAbs or TgAbs [15]. In our study, no relationship was observed between FCRL2,3,4 mRNA expression levels and TPOAbs, TgAbs and TSHRAbs, which was documented by the use of two different statistical tests. Our results suggest once again the lack of an association between FCRL expression and the immune process in GD and/or GO. It should be noted that TSHRAbs may be either thyroid-stimulating immunoglobulin (TSI) or thyroid-blocking immunoglobulin (TBI). There is a possibility that, if TSI or TBI were measured in bioassay, there may be correlation in the FCRL expression with stimulating TSHRAbs, i.e., TSI. However, considering that, generally, the prevailing pool of TSHRAbs does constitute TSI in patients with GD and that all our patients were hyperthyroid on admission (and at the time of blood collection), one can assume that they had TSI antibodies (either exclusively or in dominance). Nevertheless, this potential association of TSI/TBI with FCRL expression should be investigated in future studies.

Regarding the other potential mechanism, our research has shown a completely new relationship between FCRL4 mRNA expression and hyperthyroidism. Such results have not been published before; there were only studies concerning the relationship between FCRL3 polymorphism and thyroid function. These studies did not show any relationship between FCRL3 polymorphism and TSH/FT3 levels in patients with GD [15] or GO [20]. Nonetheless, in another study, FCRL3 expression levels in HT were observed to be increased in hypothyroid patients [18], but L-thyroxine replacement did not seem to offset the increase in FCRL3 expression; thus, authors hypothesized that the thyroid immune injury, rather than biochemical hypothyroidism, has been associated with altered FCRL3 expression in HT [18]. Thus, our results indicate that FCRL mRNA expression is associated with perturbed function of the thyroid rather than with autoimmunity in GD patients. In fact, most of the GD patients had hyperthyroidism upon admission to our department, but the majority of GO patients had euthyroid issues due to antithyroid drug treatment.

It is known that hyperthyroidism may cause changes in a number of hematological parameters. However, no statically significant differences were found in either WBC or lymphocyte concentrations between groups in our study. The lack of expected differences may be due to the relatively low number of patients in particular groups as well as the fact that WBC is affected by different factors and, therefore, only relative changes in WBC count can be of value (but we measured all parameters only at one time point). It should be stressed that only leucopenia in patients with thyrotoxicosis may be considered as being caused by raised thyroid hormone levels, which was not the case regarding our patients.

In hyperthyroidism due to GD, the percentage of T cells is significantly lower and the percentage of B-cells is higher [22]. That is why it can be hypothesized that hyperthyroidism could be a factor favoring the presence of B-cells, among other B-cells, expressing FCRL4 in peripheral blood. The previous studies have already shown a relationship between thyroid hormones and B-cells. Results of a study by Wang et al. (2023) indicate that high levels of T3 can induce abnormal differentiation and activation of B-cells and provide new insights into the role of thyroid hormones in the pathogenesis of GD [23]. It can be hypothesized that high concentrations of thyroid hormones may be one of the factors that intensify the immune process in the thyroid gland by increasing the FCRL4 positive B-cells in the blood. This may also explain the beneficial effect of antithyroid drugs on the course of the immune process in the thyroid in GD patients.

FCRLs are immunomodulatory molecules, and they have been shown to be modulated in various disease states. The limited expression of FCRL4 in peripheral blood suggests that a certain mechanism could accumulate positive FCRL4 vs. negative FCRL4 memory B-cells [6]. The presence of memory B-cells with expression of FCRL4 in the blood has been described in different pathologies associated with chronic activation of the immune system [24]. In case of HIV-infected patients with high viremia or patients with rheumatoid arthritis, chronic immune activation could induce the generation of FCRL4 B-cells in additional sites where they are not usually present, including blood or synovial fluid [24,25,26]. Hyperthyroidism can be considered as a form of inflammation caused by the systemic effects of increased thyroid hormones and, in this sense, can lead to an increase in FCRL4 B-cells in peripheral blood.

FCRLs might interplay with different thyroid conditions and may contribute to both autoimmune thyroid diseases and tumorigenesis. High expression of most FCRL genes is associated with a protective effect in many cancers [27]. However, alterations in FCRL family genes, particularly through amplification and mutation, are common in cancers [27]. The dysregulation of FCRL proteins could contribute to the survival and clonal expansion of abnormal B cells, potentially leading to the development of lymphomas in the thyroid gland. Additionally, FCRL proteins, by modulating the immune response, may indirectly influence this inflammatory environment, and chronic inflammation in the thyroid can create a microenvironment conducive to malignant transformation.

On the basis of our results, we can assume that the increased expression of the FCRL4 gene in GD patients is rather a result than a cause of GD, with a leading role of this phenomenon being hyperthyroidism. In this context, FCRL gene expression should be evaluated in future studies on patients with other forms of thyrotoxicosis, such as, e.g., toxic nodular goiter or iatrogenic thyrotoxicosis.

The scientific research in medicine aims to find its application in practice. The aberrant expression of FCRL4 in hyperthyroid GD patients could be a new diagnostic marker and a potential therapeutic marker. The relationship between mRNA FCRL4 expression level and GD course or GD prognosis seems to be possible and requires further investigation. If the results, such as those in the present study, can not directly contribute to explaining the pathogenesis of GD and GO and can not be directly used as diagnostic tools, they may be somewhat helpful to broadening the knowledge on mechanisms of thyroid dysfunction effects on the whole organism and, in such a way, may constitute a basis for future studies. Our study does constitute a step to broaden the field of immune and hormonal crosstalk as well as to confirm the role of endocrine imbalance in autoimmune diseases.

The relatively small number of patients and controls could be considered as a limitation of our study; therefore, the relationship between FCRLs and thyroid function needs to be interpreted cautiously. Additional investigations with larger sample sizes are required to explore the correlation between FCRL4 and thyroid function. Another limitation is the fact that Control individuals were younger than patients from the examined GO group. The reason can be that the frequency of some autoimmune diseases, e.g., Hashimoto’s disease, increases with age, which may be related to the influence of environmental factors in genetically predisposed individuals. Because the patients from the control group had to be healthy individuals without a history of any autoimmune diseases, cancers or any other chronic diseases, the mean age of these healthy patients meeting our criteria is lower than that of the studied group. Although a statistically significant difference was found between GO and controls regarding mean age (mean age of controls—40.56 years; mean age of GO patients—50.00 years), it does not seem to affect our main results, i.e., FCRL levels. Such an assumption is justified by the fact that GD patients, who had very similar mean age (mean age of GD—47.09 years) to GO patients, had increased FCRL levels compared to both controls and GO patients.

## 5. Conclusions

In conclusion, our findings indicate that FCRL3 and FCRL4 expression levels are significantly increased in patients with GD and that this phenomenon is associated with hyperthyroidism but not with thyroid autoimmunity. To our knowledge, this is the first investigation to show that the expression levels of FCRL genes relate to thyroid function in GD patients.

## Figures and Tables

**Table 1:** Mean values of clinical/laboratory parameters in control individuals vs. patients with Graves’ Disease (GD) vs. all patients with Graves’ Orbitopathy (GO), as well as also separately vs. patients with active Graves’ Orbitopathy (active GO) and vs. patients with inactive Graves’ Orbitopathy (inactive GO).

	Control (*n* = 16)	Patients with GD (*n* = 24)	Patients with GO (*n* = 49)	Patients with Active GO (*n* = 37)	Patients with Inactive GO (*n* = 12)
**Age [years]**	40.56 ± 3.12 *n* = 16	47.09 ± 3.10 *n* = 23 *p* = 0.160	50.00 ± 2.13 *n* = 49 *p* = 0.026	49.92 ± 2.38 *n* = 37 *p* = 0.029	50.25 ± 4.81 *n* = 12 *p* = 0.090
**Body mass [kg]**	66.98 ± 9.26 *n* = 7	60.04 ± 2.84 *n* = 11 *p* = 0.404	71.02 ± 3.63 *n* = 20 *p* = 0.625	65.88 ± 3.30 *n* = 14 *p* = 0.888	83.08 ± 7.74 *n* = 6 *p* = 0.218
**BMI [kg/m^2^]**	23.54 ± 3.29 *n* = 7	22.26 ± 0.98 *n* = 11 *p* = 0.662	26.00 ± 1.12 *n* = 20 *p* = 0.367	24.36 ± 1.02 *n* = 14 *p* = 0.762	29.82 ± 2.35 *n* = 6 *p* = 0.161
**TSH [** **mIU/L]**	1.48 ± 0.17 *n* = 16	0.016 ± 0.003 *n* = 24 *p* < 0.001	3.54 ± 1.52 *n* = 49 *p* = 0.444	2.23 ± 1.12 *n* = 37 *p* = 0.663	7.59 ± 5.17 *n* = 18 *p* = 0.182
**FT3 [pg/mL]**	3.21 ± 0.17 *n* = 13	13.01 ± 1.54 *n* = 23 *p* < 0.001	4.77 ± 0.66 *n* = 49 *p* = 0.235	4.55 ± 0.60 *n* = 37 *p* = 0.201	5.46 ± 2.02 *n* = 12 *p* = 0.261
**FT4 [ng/dL]**	1.25 ± 0.04 *n* = 14	3.75 ± 0.454 *n* = 23 *p* < 0.001	1.52 ± 0.17 *n* = 49 *p* = 0.387	1.43 ± 0.13 *n* = 37 *p* = 0.43	1.84 ± 0.56 *n* = 12 *p* = 0.267
**TPOAb [IU/mL]**	10.54 ± 0.97 *n* = 11	197.94 ± 28.74 *n* = 20 *p* < 0.001	156.44 ± 25.95 *n* = 45 *p* = 0.008	171.09 ± 30.64 *n* = 34 *p* = 0.005	111.19 ± 47.68 *n* = 11 *p* = 0.048
**TgAb [IU/mL]**	21.08 ± 3.77 *n* = 7	261.97 ± 89.16 *n* = 19 *p* = 0.119	486.81 ± 168.83 *n* = 45 *p* = 0.286	343.51 ± 162.18 *n* = 34 *p* = 0.377	929.73 ± 467.76 *n* = 11 *p* = 0.145
**TSHRAb [IU/L]**	0.44 ± 0.09 *n* = 9	13.19 ± 2.38 *n* = 24 *p* = 0.003	15.01 ± 1.98 *n* = 49 *p* = 0.003	15.52 ± 2.16 *n* = 37 *p* = 0.001	13.42 ± 4.73 *n* = 12 *p* = 0.029
**TChol [mg/dL]**	159.25 ± 15.96 *n* = 4	185.37 ± 8.43 *n* = 19 *p* = 0.203	173.00 ± 15.29 *n* = 23 *p* = 0.719	180.40 ± 19.67 *n* = 15 *p* = 0.601	159.12 ± 24.91 *n* = 8 *p* = 0.989
**HDLC [mg/dL]**	56.50 ± 10.15 *n* = 4	51.26 ± 3.24 *n* = 19 *p* = 0.537	51.87 ± 3.76 *n* = 23 *p* = 0.645	56.13 ± 4.12 *n* = 15 *p* = 0.970	43.87 ± 7.10 *n* = 8 *p* = 0.382
**LDLC [mg/dL]**	104.52 ± 6.53 n = 3	105.79 ± 7.11 *n* = 19 *p* = 0.929	101.27 ± 10.19 *n* = 22 *p* = 0.926	104.43 ± 13.83 *n* = 14 *p* = 0.990	95.75 ± 15.12 *n* = 8 *p* = 0.708
**TGs [mg/dL]**	82.00 ± 25.00 *n* = 3	126.18 ± 12.48 *n* = 17 *p* = 0.180	115.75 ± 12.26 *n* = 16 *p* = 0.283	98.80 ± 10.95 *n* = 10 *p* = 0.497	144.00 ± 24.24 *n* = 6 *p* = 0.096
**Glucose [mg/dL]**	92.25 ± 6.02 *n* = 4	86.43 ± 1.43 *n* = 23 *p* = 0.174	86.50 ± 1.88 *n* = 26 *p* = 0.289	87.44 ± 2.53 *n* = 16 *p* = 0.421	85.00 ± 2.87 *n* = 10 *p* = 0.326
**RBC [10^12^/L]**	4.92 ± 0.13 *n* = 4	4.65 ± 0.08 *n* = 24 *p* = 0.264	4.52 ± 0.08 *n* = 30 *p* = 0.107	4.41 ± 0.11 *n* = 20 *p* = 0.071	4.67 ± 0.13 *n* = 10 *p* = 0.305
**Hgb [g/dL]**	14.60 ± 0.62 *n* = 4	13.71 ± 0.26 *n* = 24 *p* = 0.202	13.00 ± 0.23 *n* = 30 *p* = 0.026	13.09 ± 0.28 *n* = 20 *p* = 0.038	12.83 ± 0.45 *n* = 10 *p* = 0.050
**WBC [10^9^/L]**	7.07 ± 0.44 *n* = 4	7.07 ± 0.43 *n* = 24 *p* = 0.915	7.46 ± 0.28 *n* = 30 *p* = 0.625	7.63 ± 0.30 *n* = 20 *p* = 0.435	7.12 ± 0.59 n = 10 *p* = 0.964
**Neutrophils** **[10^9^/L]**	3.78 ± 0.79 *n* = 4	3.49 ± 0.25 *n* = 24 *p* = 0.679	4.01 ± 0.79 *n* = 30 *p* = 0.734	4.05 ± 0.27 *n* = 20 *p* = 0.703	3.91 ± 0.34 *n* = 10 *p* = 0.871
**Lymphocytes** **[10^9^/L]**	2.42 ± 0.43 *n* = 4	2.62 ± 0.16 *n* = 24 *p* = 0.644	2.51 ± 0.13 *n* = 30 *p* = 0.819	2.56 ± 0.14 *n* = 20 *p* = 0.706	2.41 ± 0.27 *n* = 10 *p* = 0.975
**Eosinophils** **[10^9^/L]**	0.263 ± 0.05 *n* = 4	0.351 ± 0.117 *n* = 24 *p* = 0.764	0.23 ± 0.03 *n* = 30 *p* = 0.762	0.22 ± 0.02 *n* = 20 *p* = 0.560	0.24 ± 0.11 *n* = 10 *p* = 0.920
**Basophils** **[10^9^/L]**	0.057 ± 0.011 *n* = 4	0.079 ± 0.036 *n* = 24 *p* = 0.806	0.093 ± 0.027 *n* = 30 *p* = 0.666	0.099 ± 0.003 *n* = 20 *p* = 0.623	0.073 ± 0.042 *n* = 10 *p* = 0.805
**Monocytes** **[10^9^/L]**	0.550 ± 0.04 *n* = 4	0.646 ± 0.051 *n* = 24 *p* = 0.461	0.62 ± 0.04 *n* = 29 *p* = 0.532	0.69 ± 0.05 *n* = 20 *p* = 0.203	0.460 ± 0.05 *n* = 9 *p* = 0.293
**ASPAT [U/L]**	24.50 ± 1.04 *n* = 4	26.75 ± 1.72 *n* = 24 *p* = 0.604	27.45 ± 2.00 *n* = 29 *p* = 0.594	27.58 ± 2.61 *n* = 19 *p* = 0.602	27.20 ± 3.19 *n* = 10 *p* = 0.613
**ALAT [U/L]**	26.75 ± 3.64 *n* = 4	35.87 ± 2.78 *n* = 24 *p* = 0.206	26.83 ± 2.44 *n* = 29 *p* = 0.991	28.53 ± 3.41 *n* = 19 *p* = 0.82	23.60 ± 2.82 *n* = 10 *p* = 0.545
**Vit D [ng/mL]**	19.20 ± 6.10 *n* = 3	19.40 ± 1.78 *n* = 18 *p* = 0.968	19.20 ± 2.86 *n* = 14 *p* = 0.813	21.25 ± 3.76 *n* = 10 *p* = 0.794	19.82 ± 4.29 *n* = 4 *p* = 0.934
**CRP**	-	0.70 ± 0.27 *n* = 18	0.64 ± 0.32 *n* = 21	0.59 ± 0.27 *n* = 14	0.73 ± 0.42 *n* = 7

Comparison between subgroups was performed by Student’s unpaired *t*-test. Statistical significance was determined at the level of *p* < 0.05. Statistically significant differences between individual examined groups and Control are shaded. Other statistical differences between individual examined groups are specified in the Section 3.

**Table 2:** Univariate logistic regression of active GO determinants (active vs. inactive GO) (linear variables) performed in the group of patients with GO comprising active GO + inactive GO.

Variable	Patients with Active GO + Inactive GO *n* = 37 + 12 = 49		
	OR	95 %Cl	*p*
**FCRL2**	1.453	2.938	0.299
**FCRL3**	1.117	2.520	0.790
**FCRL4**	1.123	1.546	0.475

Only such variables as FCRLs are presented in the univariate analysis. Such variables as thyroid tests (TSH, FT3 and FT4) and thyroid antibodies were not put into statistical analysis as they are directly associated with dichotomized variables, i.e., active GO. Other linear variables are not presented as they were not statistically significant. Statistical significance was determined at the level of *p* < 0.05. OR, odds ratio, 95% CI, 95% confidence interval. *p*, level of statistical significance.

**Table 3:** Univariate logistic regression analysis of hyperthyroidism determinants (linear variables) performed in the following groups: the whole group of individuals (*n* = 89) (above), the whole group of individuals after excluding hypothyroid patients (*n* = 89 − 7 = 82) (below).

**Variable**	**The Whole Group of Individuals** ***n* = 89**		
	**OR**	**95% Cl**	***p***
**FCRL2**	0.987	1.422	0.942
**FCRL3**	1.148	1.837	0.564
**FCRL4**	1.319	1.654	0.016
**Variable**	**The Whole Group of Individuals** **after Excluding Hypothyroid Patients** ***n* = 82**		
	**OR**	**95% Cl**	***p***
**FCRL2**	1.114	1.730	0.632
**FCRL3**	1.322	2.344	0.339
**FCRL4**	1.561	2.213	0.012

Only such variables as FCRLs are presented in the univariate analysis. Such variables as thyroid tests (TSH, FT3 and FT4) and thyroid antibodies were not put into statistical analysis, as they are directly associated with a dichotomized variable, i.e., hyperthyroidism. Other linear variables are not presented as they were not statistically significant. Statistical significance was determined at the level of *p* < 0.05. Statistically significant differences are shaded. OR, odds ratio, 95%CI, 95% confidence interval. *p*, level of statistical significance.

**Table 4:** Median of relative mRNA expression of particular FCRL subtypes (FCRL2, FCRL3, FCRL4) in patients with positive vs. negative thyroid antibodies (TPOAb (+) vs. TPOAb (−); TgAb (+) vs. TgAb (−); TSHRAb (+) vs. TSHRAb (−)).

**GD**	**TPOAb (+)**	**TPOAb (−)**	**TgAb (+)**	**TgAb (−)**	**TSHRAb (+)**	**TSHRAb (−)**
**FCRL2**	1.522 *n* = 21	1.883 *n* = 2 *p* = 0.864	1.298 *n* = 9	1.477 *n* = 10 *p* = 0.438	-	-
**FCRL3**	1.123 *n* = 17	1.535 *n* = 2 *p* = 0.947	0.840 *n* = 8	1.752 *n* = 10 *p* = 0.120	-	-
**FCRL4**	2.508 *n* = 13	6.576 *n* = 2 *p* = 0.445	2.344 *n* = 6	3.073 *n* = 8 *p* = 0.414	-	-
**GO**	**TPOAb (+)**	**TPOAb (−)**	**TgAb (+)**	**TgAb (−)**	**TSHRAb (+)**	**TSHRAb (−)**
**FCRL2**	0.716 *n* = 29	0.852 *n* = 16 *p* = 0.530	0.832 *n* = 15	0.761 *n* = 29 *p* = 0.824	0.739 *n* = 45	0.963 *n* = 4 *p* = 0.352
**FCRL3**	0.667 *n* = 29	0.886 *n* = 16 *p* = 0.824	0.783 *n* = 14	0.660 *n* = 29 *p* = 0.525	0.663 *n* = 44	1.340 *n* = 4 *p* = 0.198
**FCRL4**	1.178 *n* = 28	0.937 *n* = 14 *p* = 0.480	1.186 *n* = 13	0.937 *n* = 28 *p* = 0.293	1.127 *n* = 43	1.409 *n* = 3 *p* = 0.449
**GD + GO**	**TPOAb (+)**	**TPOAb (−)**	**TgAb (+)**	**TgAb (−)**	**TSHRAb (+)**	**TSHRAb (−)**
**FCRL2**	0.829 *n* = 46	0.944 *n* = 19 *p* = 0.880	0.832 *n* = 24	0.826 *n* = 39 *p* = 0.721	0.832 *n* = 69	0.963 *n* = 4 *p* = 0.619
**FCRL3**	0.888 *n* = 45	0.788 *n* = 18 *p* = 0.257	0.888 *n* = 22	0.794 *n* = 39 *p* = 0.988	0.886 *n* = 67	1.340 *n* = 4 *p* = 0.509
**FCRL4**	1.956 *n* = 40	1.003 *n* = 17 *p* = 0.480	1.631 *n* = 19	1.451 *n* = 36 *p* = 0.555	1.956 *n* = 62	1.409 *n* = 3 *p* = 0.888

GD, Graves’ Disease; GO, Graves’ Orbitopathy; TPOAb, thyroid peroxidase antibody; TgAb, thyroglobulin antibody; TSHRAb, TSH receptor antibody; n, number of patients; *p*, level of statistical significance. Mann–Whitney test was used for statistical comparison between medians of particular groups. All patients with GD had positive TSHRAbs; therefore, it was impossible to compare them to patients with TSHRAbs (−). Statistical significance was determined at the level of *p* < 0.05.

**Table 5:** Univariate logistic regression analysis of thyroid antibody positivity determinants (linear variables) performed in the whole group of individuals.

**Variable**	**The Whole Group of Individuals *n* = 89** **TPOAb**		
	**OR**	**95% Cl**	***p***
**FCRL2**	1.083	1.605	0.690
**FCRL3**	1.454	2.664	0.226
**FCRL4**	1.180	1.459	0.127
**Variable**	**The Whole Group of Individuals *n* = 89** **TgAb**		
	**OR**	**95% Cl**	***p***
**FCRL2**	0.908	1.370	0.646
**FCRL3**	0.763	1.436	0.402
**FCRL4**	1.053	1.184	0.383
**Variable**	**The Whole Group of Individuals *n* = 89** **TSHRAb**		
	**OR**	**95% Cl**	***p***
**FCRL2**	0.996	1.618	0.989
**FCRL3**	1.811	1.811	0.964
**FCRL4**	1.09	1.440	0.435

Statistical analyses were performed separately for positive TPOAbs (above), positive TgAbs (middle), positive TSHRAbs (below). Only results (not statistically significant) for FCRL relative expression are presented in tables. Such variables as thyroid tests (TSH, FT3 and FT4) were not put into statistical analyses, as they are directly associated with a dichotomized variable, i.e., thyroid antibody positivity. Statistical significance was determined at the level of *p* < 0.05. OR, odds ratio, 95%CI, 95% confidence interval. *p*, level of statistical significance.

## Data Availability

The raw data supporting the conclusions of this article will be made available by the authors without undue reservation.

## References

[1] Popa O., Balaș M., Golu I., Amzăr D., Varcuș F., Cornianu M., Iacob M., Popa V.T., Vlad M.J. (2024). Medullary Thyroid Carcinoma in Patients with Graves’ Disease-A Case Series and Literature Review. J. Clin. Med..

[2] Mkaddem S.B., Benhamcu B., Monteiro R.C. (2019). Understanding Fc receptor involvement in inflammatory disease: From mechanism to new therapeutic tools. Front. Immunol..

[3] Davis R.S. (2007). Fc receptor-like molecules. Annu. Rev. Immunol..

[4] Mamidi M.K., Huang J., Honjo K., Li R., Tabengwa E.M., Neeli I., Randall N.L., Ponnuchetty M.V., Radic M., Leu C.M. (2023). FCRL1 immunoregulation in B-cell development and malignancy. Front. Immunol..

[5] Rostamzadeh D., Kazemi T., Amirghofran Z., Shabani M. (2018). Update on Fc receptor-like (FCRL) family: New immunoregulatory players in health and diseases. Expert. Opin. Ther. Targets.

[6] Ehrhardt G.R., Cooper M.D. (2011). Immunoregulatory roles for fc receptor-like molecules. Curr. Top. Microbiol. Immunol..

[7] Matos M.C., Pinheiro A., Melo-Ferreira J., Davis R.S., Esteves P.J. (2021). Evolution of Fc Receptor-like scavenger in mammals. Front. Immunol..

[8] Gauld S.B., Dal Porto J.M., Cambier J.C. (2002). B-cell antigen receptor signaling: Roles in cell development and disease. Science.

[9] Shlapatska L.M., Mikhalap S.V., Berdova A.G., Zelensky O.M., Yun T.J., Nichols K.E., Clark E.A., Sidorenko S.P. (2001). CD150 association with either the SH2-containig inositol phosphatase or SH2-containing protein tyrosine phosphatase is regulated by the adaptor protein SH2D1A. J. Immunol..

[10] Kochi Y., Myouzen K., Yamada R., Suzuki A., Kurosaki T., Nakamura Y., Yamamoto K. (2009). Affiliations FCRL3, an autoimmune susceptibility gene, has inhibitory potential on B-cell receptor-mediated signaling. J. Immunol..

[11] Rostamzadeh D., Dabbaghmanesh M.H., Shabani M., Hosseini A., Amirghofran Z. (2015). Expression profile of human Fc receptor-like1, 2, and 4 molecules in peripheral blood mononuclear cells of patients with Hashimoto’s thyroiditis and Graves ‘disease. Horm. Metab. Res..

[12] Zhao S.X., Liu W., Zhan M., Song Z.Y., Yang S.Y., Xue L.Q., Pan C.M., Gu Z.H., Liu B.L., Wang H.N. (2013). A refined study of FCRL genes from a genome-wide association study for Graves’ disease. PLoS ONE.

[13] Wojciechowska-Durczynska K., Wieczorek-Szukala K., Stefanski B., Zygmunt A., Stepniak J., Karbownik-Lewinska M., Lewinski A. (2021). Percentage of myeloid dendritic cells in peripheral venous blood is negatively related to incidence of Graves’ orbitopathy. Mediat. Inflamm..

[14] Bartalena L., Kahaly G.J., Baldeschi L., Dayan C.M., Eckstein A., Marcocci C., Marinò M., Vaidya B., Wiersinga W.M. (2021). The 2021 European Group on Graves’ orbitopathy (EUGOGO) clinical practice guidelines for the medical management of Graves’ orbitopathy. Eur. J. Endocrinol..

[15] Jin G.X., Zhou Y.Y., Lei Y., Bi Y.X. (2015). Correlation between single nucleotide polymorphism of FCRL-3 gene and Graves’ disease in Han population of northern Anhui province, China. Int. J. Clin. Exp. Med..

[16] Oeverhaus M., Sander J., Smetana N., Bechrakis N.E., Inga N., Al-Ghazzawi K., Chen Y., Eckstein A. (2024). How Age Affects Graves’ Orbitopathy-A Tertiary Center Study. J. Clin. Med..

[17] Cieplińska K., Niedziela E., Kowalska A. (2023). Immunological Processes in the Orbit and Indications for Current and Potential Drug Targets. J. Clin. Med..

[18] Štefanić M., Tokić S., Suver-Stević M., Glavaš-Obrovac L. (2019). Expression of TIGIT and FCRL3 is altered in T Cells from patients with distinct patterns of chronic autoimmune thyroiditis. Exp. Clin. Endocrinol. Diabetes.

[19] Wojciechowska-Durczynska K., Krawczyk-Rusiecka K., Zygmunt A., Stawerska R., Lewinski A. (2016). In children with autoimmune thyroiditis CTLA4 and FCRL3 genes–but not PTPN22–are overexpressed when compared to adults. Neuro Endocrinol. Lett..

[20] Wu S., Cai T., Chen F., He X., Cui Z. (2015). Genetic associations of FCRL3 polymorphisms with the susceptibility of Graves ophthalmopathy in a Chinese population. Int. J. Clin. Exp. Med..

[21] Capone M., Bryant J.M., Sutkowski N., Haque A. (2016). Fc Receptor-Like Proteins in Pathophysiology of B-cell Disorder. J. Clin. Cell Immunol..

[22] Mori H., Amino N., Iwatani Y., Kabutomori O., Asari S., Motoi S., Miyai K., Kumahara Y. (1980). Increase of peripheral B lymphocytes in Graves’ disease. Clin. Exp. Immunol..

[23] Wang J., Li G.Q., Liu S., Miao J.J., Sun Q., Gu W.S., Mao X.M. (2023). Activation of Toll-like receptor 4 by thyroid hormone triggers abnormal B-cell activation. Immun. Inflamm. Dis..

[24] Jourdan M., Robert N., Cren M., Thibaut C., Duperray C., Kassambara A., Cogné M., Tarte K., Klein B., Moreaux J. (2017). Characterization of human FCRL4-positive B-cells. PLoS ONE.

[25] Yeo L., Lom H., Juarez M., Snow M., Buckley C.D., Filler A., Raza K., Scheel-Toellner D. (2015). Expression of FcRL4 defines a pro-inflammatory, RANKL-producing B-cell subset in rheumatoid arthritis. Ann. Rheum. Dis..

[26] Siewe B., Nipper A.J., Sohn H., Stapleton J.T., Landay A. (2017). FcRL4 expression identifies a pro-inflammatory B-cell subset in viremic HIV-infected subjects. Front. Immunol..

[27] Liang X., Du L., Fan Y. (2023). The potential of FCRL genes as targets for cancer treatment: Insights from bioinformatics and immunology. Aging.

